# Evaluation of the Surface Characteristics of Dental CAD/CAM Materials after Different Surface Treatments

**DOI:** 10.3390/ma13040981

**Published:** 2020-02-22

**Authors:** Konstantinos Papadopoulos, Kimon Pahinis, Kyriaki Saltidou, Dimitrios Dionysopoulos, Effrosyni Tsitrou

**Affiliations:** 1 Department of Operative Dentistry, Faculty of Dentistry, School of Health Sciences, Aristotle University of Thessaloniki, Thessaloniki 54124, Greece; cospapadopoulos@hotmail.com (K.P.); etsitrou@dent.auth.gr (E.T.); 2 A Department of Dentistry, Nea Moudania National Health Center, Nea Moudania 63200, Greece; k.pahinis@gmail.com; 3 Department of Chemistry, Aristotle University of Thessaloniki, Thessaloniki 54124, Greece; saltidoukyriaki@gmail.com

**Keywords:** CAD/CAM materials, hydrofluoric acid etching, optical profilometry, sandblasting, scanning electron microscopy, surface roughness, surface treatment

## Abstract

Computer-aided design/computer-aided manufacturing (CAD/CAM) technology was developed to ensure the sufficient strength of tooth restorations, to improve esthetic restorations with a natural appearance and to make the techniques easier, faster and more accurate. In the view of the limited research on the surface treatments of the CAD/CAM materials and the need to evaluate the ideal surface characteristics of a material to achieve the best adhesion to tooth tissues, this study aimed to investigate the surface roughness and morphology of four different CAD/CAM materials using four different surface treatments. The CAD/CAM materials used in this study were three composites (Shofu Block HC, Lava Ultimate and Brilliant Crios) and a hybrid ceramic (Enamic). The surface of the specimens of each material received one of the following treatments: no surface treatment, sandblasting with 29 μm Al_2_O_3_ particles, 9% hydrofluoric acid etching and silane application, and the tribochemical method using CoJet System. Surface roughness was evaluated using optical profilometry, and surface morphology was observed by means of scanning electron microscopy. All surface treatments resulted in higher surface roughness values compared to the control group. Different treatments affected the surface properties of the materials, presumably due to discrepancies in their composition and structure.

## 1. Introduction

Digital dentistry has been recently introduced and has become a new challenge for dental practitioners. Computer-aided design/computer-aided manufacturing (CAD/CAM) technology is broadly used in daily dental practice due to its advantages such as its speed, ease of use, and quality of therapy [1]. This technology can be used in both the dental laboratory and the dental office with multiple applications which include the fabrication of indirect restorations (inlays, onlays, veneers and crowns), fixed partial dentures, implant abutments, full-mouth reconstruction and orthodontics [1,2]. CAD/CAM technology was developed in order to ensure the sufficient strength of tooth restorations, to improve esthetic restorations with a natural appearance and to make the techniques easier, faster and more accurate [3]. 

More specifically, digital scans of the denture provide faster and easier treatment in comparison with the conventional impressions because casts, wax-ups, investing, casting and firing are eliminated. Moreover, having a milling machine on-site means that patients can receive their permanent restorations at the first appointment without the need to have provisional restorations, which take time to fabricate and fit. The quality of CAD/CAM restorations is high because measurements and fabrication are precise due to the applied digital technology [4]. On the other hand, there are also some disadvantages of CAD/CAM technology; most important among these are the initial cost of the equipment and software, and the need to spend time and money on training [2].

Indirect tooth restorations are a very common indication for dental CAD/CAM technology [5]. The CAD/CAM materials of choice for this type of restorations are either ceramic or composite. Recently, there has been an evolution of CAD/CAM composite materials due to their improved physical and mechanical properties in comparison to their ceramic counterparts, which were achieved by changes in their manufacturing methods (high pressure, high temperature) and structure (glass ceramic networks) [6,7].

CAD/CAM composites present less hardness and stiffness compared to ceramics, and as a result, the opposing tooth tissues are subjected to less wear clinically and they are friendlier to the milling machine. Furthermore, composites are easily fabricated and repaired and they are less brittle than ceramics [8], leading to less chipping and crack formation during manufacturing [9] and to improved marginal quality (potential thickness up to 0.2 mm) [10,11].

CAD/CAM composites can be classified based on their microstructural geometry into two main categories: a) resin with dispersed fillers and b) polymer-infiltrated ceramic networks (PICN) [12]. The first category includes composite blocks containing a basic monomer type (bisphenol A diglycidylmethacrylate (Bis-GMA), urethane dimethacrylate (UDMA), triethylene glycol dimethacrylate (TEGDMA), etc.) as an organic matrix with dispersed filler particles (silica, zirconia, barium glass, etc.) [5]. PICN materials consist of a three-dimensional ceramic network which is infiltrated with a monomer mixture, offering a higher Weibull modulus and making the material less brittle than glass ceramics [13,14].

Indirect restorations made by CAD/CAM composite materials are bonded to the tooth surfaces using resin cements. The increase of the bond strength between indirect CAD/CAM restorations and resin cement is essential to improve fracture resistance and to preserve the marginal integrity of restorations [15,16]. To achieve an adequate bond to CAD/CAM composite restorative materials, mechanical or chemical pre-treatments to the bonded surfaces are necessary [17,18]. Chemical bonds between resin cement and resin-based restorative material [17], as well as the application of primers in order to wet polymeric resin surfaces, significantly enhance the adhesive bonding [19,20]. In addition, micromechanical pre-treatments mainly by using hydrofluoric acid (HF) or sandblasting with aluminum oxide (Al_2_O_3_) particles can also improve the bonding of the surfaces [21]. 

Surface modification achieved by the application of HF or sandblasting with Al_2_O_3_ particles creates a micro-retentive surface that enables the mechanical interlocking of the resin cement [22]. Additionally, a tribochemical method has been introduced comprising sandblasting with silica-coated particles instead of pure Al_2_O_3_, which leads to an additional chemical bonding [23] by incrementing the silica (SiO_2_) content in the surface in order to apply a silane prior to the adhesive agent [24].

This micromechanical interlocking of the interface between the two bonding surfaces is strongly dependent on their surface roughness and surface morphology [25]. In view of the limited research on the surface treatments of the CAD/CAM composite blocks and the need to evaluate the ideal surface characteristics of the material to achieve the best adhesion to tooth tissues, this study aimed to investigate the surface roughness and morphology of four different CAD/CAM blocks using four different surface treatments. The quantitative and qualitative evaluations were carried out using an optical profilometer and scanning electron microscopy (SEM), respectively. The first null hypothesis of this investigation was that there were no differences among the surface treatments for each CAD/CAM material in surface roughness and morphology; the second null hypothesis was that there were no differences in the same surface characteristics among the CAD/CAM materials treated with the same method.

## 2. Materials and Methods

### 2.1. Preparation of the Specimens

Four CAD/CAM materials were used in this study: three composite materials (Shofu Block HC (SH), Shofu; Lava Ultimate (LV), 3M ESPE and Brilliant Crios (BR), Coltene) and one hybrid ceramic material (Enamic (EN), Vita Zahnfabrik). The technical characteristics of the materials tested in this investigation are presented in Table 1.

The CAD/CAM blocks were sectioned in slabs of 4 mm in thickness using a water-cooled diamond saw (Isomet 11–1180 Buehler, Lake Bluff, IL, USA). Eight slabs of each material (32 slabs in total) were cut and mounted in self-cured acrylic resin (NT Newton AYCLIFFE, Antalya Turkey) prior finishing and polishing. The surfaces of the slabs were ground on a grinding machine (Jean Wirtz TG 250, Dusseldorf, Germany) with 200 rpm under water cooling (50 ml/min), using up to 1200 grit silicon carbide abrasive papers (Apex S system, Buehler, Lake Bluff, IL, USA) and then polished using a short nap synthetic cloth combined with 6 μm and 1 μm ultra-fine polishing pastes (Veltex, Meta Di Diamond suspensions, Buehler, Lake Bluff, IL, USA). After polishing, the specimens were immersed in an ultrasonic bath (Euronda Spa, Montecchio Precalcino, Vicenza, Italy) for 5 min to remove any impurities.

### 2.2. Experimental Groups of the Study

**Figure 1:**
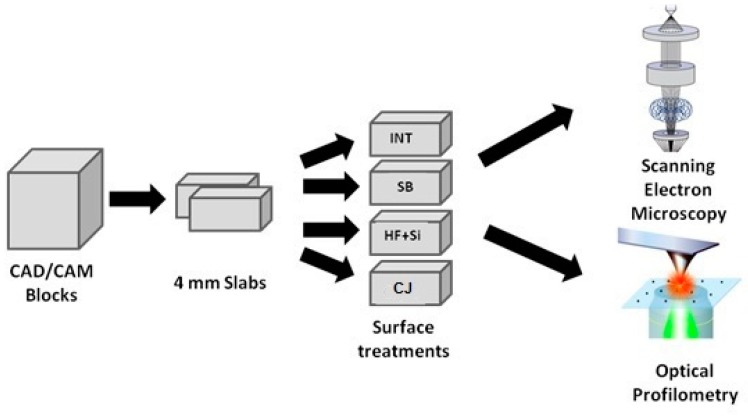
The schematic design of the study.

The eight slabs of each material were divided into four subgroups, and the top surface of each specimen received one of the following treatments (Figure 1): 

Group I: No surface treatment (INT): Specimens remained intact and served as the control of the study.

Group II: Sandblasting (SB): The top surfaces of the specimens were sandblasted using Aquacare^TM^ Twin (Velopex Int, London, UK) with 29 μm Al_2_O_3_ particles (AquaAbrasion^TM^, Velopex Int, London, UK), and the tip of the handpiece was positioned vertically to the surface from a distance of 10 mm over the course of 20 s with a pressure of 0.25 MPa. Remaining particles were removed using an air syringe.

Group III: Hydrofluoric acid etching and silane (HF + Si): The surface was etched with 9% hydrofluoric acid (Porcelain Etch, Ultradent, South Jordan, UT, USA) for 90 s and subsequently rinsed with water spray for 60 s. This step was followed by ultrasonic cleaning (distilled water, 5 min) and air dried. A silane coupling agent (Silane, Ultradent, South Jordan, UT, USA) was applied throughout the surface using a microbrush and left intact for 60 s.

Group IV: Tribochemical silica coating and silane (CJ): The surface was sandblasted using the CoJet System (CoJet, 3M, ESPE, Seefeld, Germany) with 30 μm silicazed Al_2_O_3_ particles (CoJet Sand, 3M, ESPE, Seefeld, Germany) and the tip was directed vertically to the surface at a distance of 10 mm, for a duration of 15 s and an air pressure of 2.5 bars, according to the manufacturer’s instructions. The remaining particles were removed using an air syringe. The same silane coupling agent as Group III was applied throughout the surface using a microbrush and was left intact for 60 s.

### 2.3. Evaluation of Surface Roughness

The surface roughness analysis of the specimens was implemented according to ISO 25178 (non-contact type), which is related to the analysis of 3D areal surface textures. The measurements were performed prior to and after the treatments using a Vertical Scanning Interference (VSI) microscope (Contour GTI 3D, Bruker Corp, MA, USA). One image was obtained (magnification ×20) from each specimen at the four quadrants of each specimen’s surface, corresponding to a surface of 0.317 × 0.238 mm^2^. Vision64™ software (Bruker Corp, MA, USA) was used to acquire the data and compute the arithmetical mean height of the surface (S_a_) and the maximum average between highest peaks and highest valleys of the surface (S_z_) of each image. The values of the four images of each specimen were averaged and the mean values were calculated. 

### 2.4. SEM Observations of Surface Morphology 

Aiming to investigate the changes in the surface morphology of the materials after the treatments, the specimens of each experimental group were observed using SEM. The specimens were mounted on aluminum stubs, sputter-coated with carbon to a thickness of approximately 200 Å in a vacuum evaporator (at low vacuum) and examined under SEM (JEOL Ltd, JSM-840, Tokyo, Japan) at an accelerated voltage of 20 KV. Four photomicrographs were performed (one in every quadrant) at ×1000 magnification on the surface area of the specimens in order to detect any changes in surface morphology after the treatments.

### 2.5. Statistical Analysis

The sample size was determined using the formula: $n=\frac{t_{\alpha /2}^{2}\times s^{2}}{e^{2}}$, where n = sample size, t_α/2_ = coefficient according to the t distribution, α = 0.05 (level of significance), s = standard deviation, and e = sampling error [26]. These parameters were estimated from a pilot experimental work, and it was found that n was approximately equal to eight specimens. The data were statistically analyzed using SPSS Statistics 20.0 software (IBM Corp, ILL, Chicago, USA). Surface roughness data were preliminary tested for normality and homogeneity using the Shapiro–Wilk test and Levene test, respectively, and statistically analyzed using full factorial ANOVA. Dunnett’s post-hoc test was used to detect statistical differences at a level of significance α = 0.05. 

## 3. Results

### 3.1. Surface Roughness Outcomes

**Figure 2:**
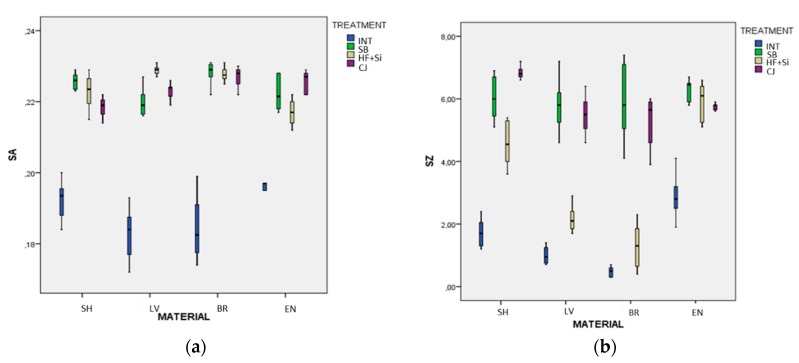
(**a**) Box plot results of the S_a_ data, (**b**) box plot results of the S_z_ data. SH: Shofu Block HC, LV: Lava Ultimate, BR: Brilliant Crios, EN: Vita Enamic, INT: intact, SB: sandblasting, HF + Si: hydrofluoric acid and silane, CJ: Cojet system.

Figure 2a,b present the S_a_ and S_z_ box plot results, respectively, clustered according to material and surface treatment. In this process, seven outliers from the initial data of 128 data observations have been removed.

Table 2 presents the means and the standard deviations of the S_a_ (μm) and S_z_ (μm) dependent variables with respect to the material and surface treatment sources. The group size (n) refers to the actual number of measurements used in the analysis after the outliers have been removed.

A full factorial analysis of variance (FFANOVA) has been used to examine the differential influence of the two sources of material and surface treatment on the two dependent variables S_a_ and S_z_, respectively. The results of the analysis are presented in Table 3. It is seen that both the main effects and the interaction effects are significant at the significant level *α* = 0.05.

The significance of the interaction effects indicates that a detailed analysis should be employed to trace the possible differences of means between groups. Moreover, considering that Levene’s test of equality of error variances supports the hypothesis that the variances between groups are different at *p* = 0.000 for both S_a_ and S_z_, we employed Dunnett’s post-hoc test as the most appropriate test for this examination. 

In particular, it was found that, for SH, the pairs of SB vs. HF + Si (*p* = 0.593) and HF + Si vs. CJ (*p* = 0.249) for S_a_, and SB vs. CJ (*p* = 0.075) for S_z_ were not significantly different; for LV, the pairs of SB vs. CJ for both S_a_ (*p* = 0.364) and for S_z_ (*p* = 0.955) were not significantly different; for BR, the pairs of SB vs. HF + Si (*p* = 0.999), SB vs. CJ (*p* = 0.939), and HF + Si vs. CJ for S_a_ (*p* = 0.987), and INT vs. HF + Si (*p* = 0.065), and SB vs. CJ (*p* = 0.747) for S_z_ were not significantly different; finally, for ENm the pairs of SB vs. HF + Si *(p =* 0.248) and SB vs. CJ *(p =* 0.730) for S_a_, and SB vs. HF + Si *(p =* 0.577), SB vs. CJ (*p* = 0.055) and HF + Si vs. CJ (*p* = 0.970) for S_z_ were not significantly different. All the other pairs of treatments for any type of material were found to differ significantly for both S_a_ and S_z_ (*p* < 0.05). 

Similarly, but reversing the interaction architecture, it was found that, for INT groups, the pairs of SH vs. BR (*p* = 0.260), SH vs. EN *(p* = 0.327), and LV vs. BR (*p* = 0.999) for S_a_ were not significantly different; for SB, the pairs of SH vs. BR (*p* = 0.368), SH vs. EN (*p* = 0.577), LV vs. EN (*p* = 0.859) and BR vs. EN (*p* = 0.160) for S_a_, and all the pairs of materials for S_z_ were not significantly different (*p >* 0.05); for HF+Si, the pairs of SH vs. LV (*p* = 0.051), SH vs. BR (*p* = 0.134), SH vs. EN (*p* = 0.089), LV vs. BR (*p* = 0.738) for S_a_, and LV vs. BR (*p* = 0.060) for S_z_ were not significantly different; finally, for CJ, the pairs of SH vs. LV (*p* = 0.059), LV vs. EN (*p* = 0.508), and BR vs. EN (*p* = 0.892) for S_a_, and LV vs. BR (*p* = 0.992), LV vs. EN (*p* = 0.848), and BR vs. EN (*p* = 0.571) for S_z_ were not significantly different. All the other pairs of materials for any type of treatment found to differ significantly for both S_a_ and S_z_ (*p* < 0.05).

**Figure 3:**
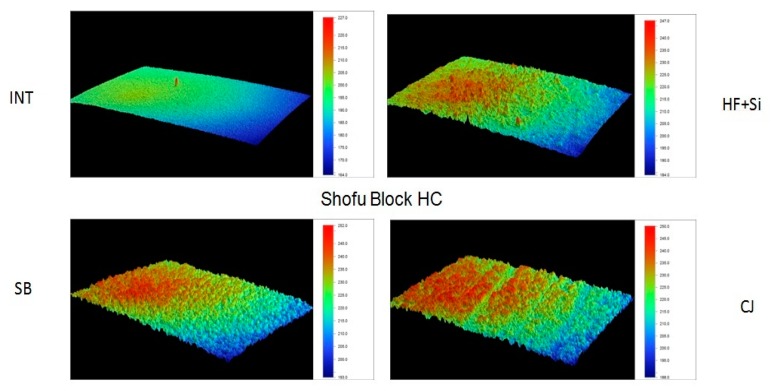
Representative 3D topographic surface maps of Shofu Block HC (×20 magnification).

**Figure 4:**
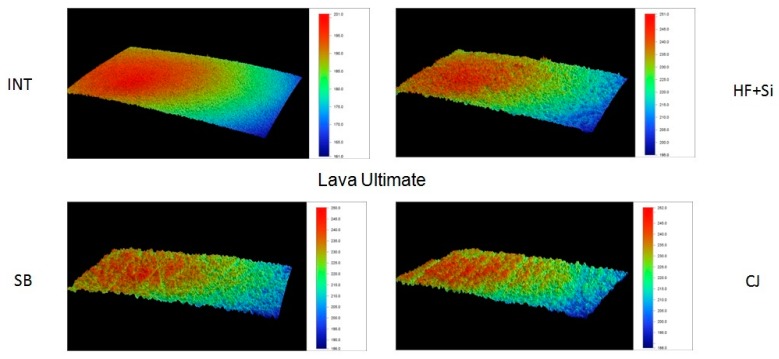
Representative 3D topographic surface maps of Lava Ultimate (×20 magnification).

**Figure 5:**
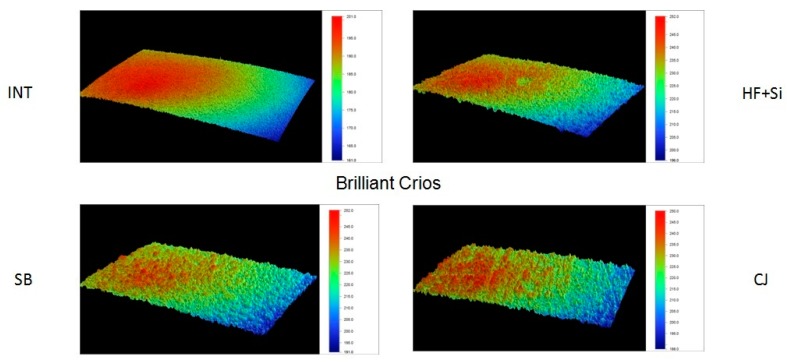
Representative 3D topographic surface maps of Brilliant Crios (×20 magnification).

**Figure 6:**
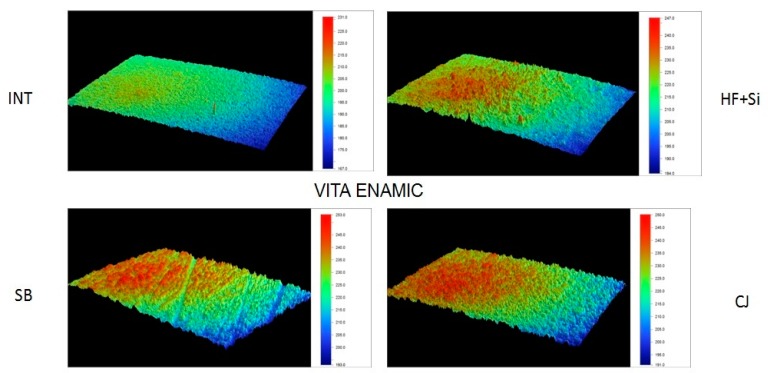
Representative 3D topographic surface maps of Vita Enamic (×20 magnification).

Representative 3D topographic surface maps at a magnification of ×20 for each experimental group of the study are illustrated in Figure 3, Figure 4, Figure 5 and Figure 6.

### 3.2. SEM Observations

**Figure 7:**
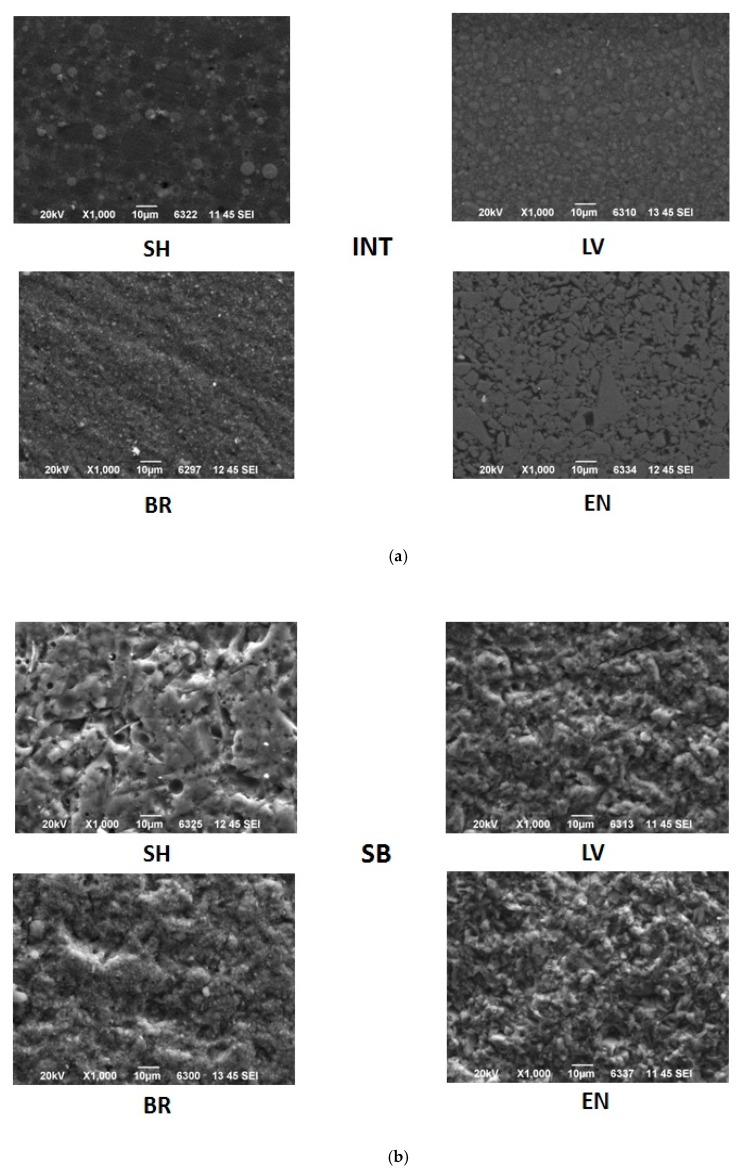

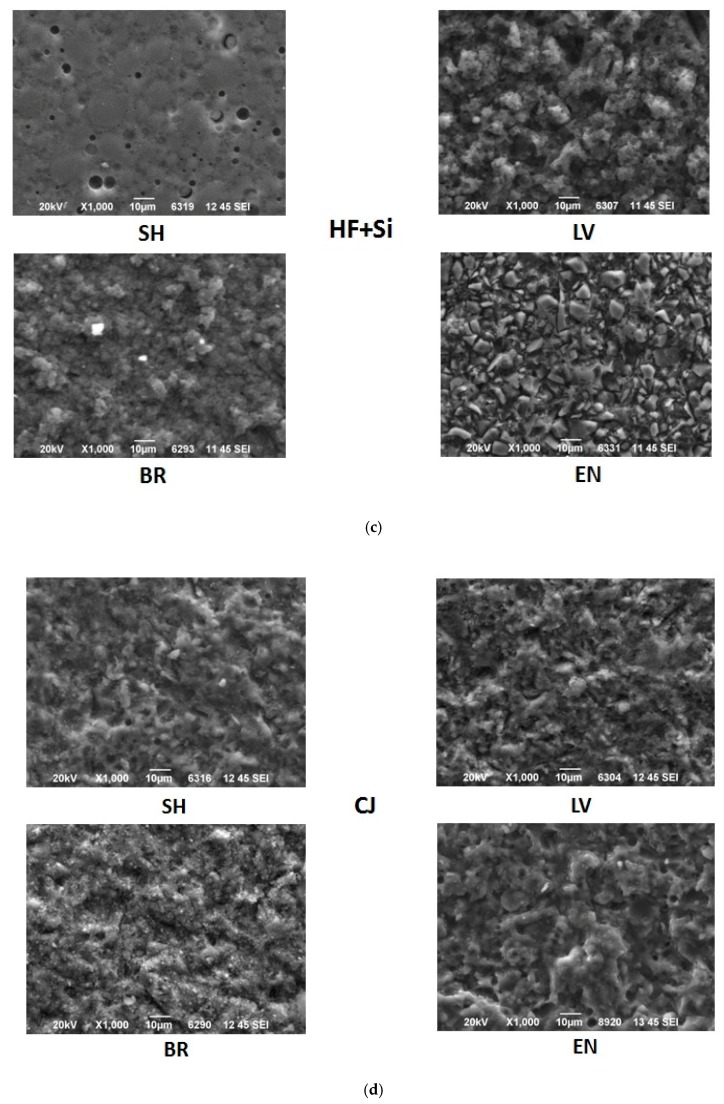
(**a**) Representative scanning electron microscope (SEM) images of the intact surfaces of the tested CAD/CAM materials. (**b**) Representative SEM images of the surfaces of the tested CAD/CAM materials after sandblasting with 29 μm Al_2_O_3_ particles. (**c**) Representative SEM images of the surfaces of the tested CAD/CAM materials after HF + Si treatment. (**d**) Representative SEM images of the surfaces of the tested CAD/CAM materials after sandblasting with the CoJet system.

Representative photomicrographs of the surface of the specimens of each experimental group of the study are illustrated in Figure 7a–d. Observations of the SEM images revealed alterations in the surface morphology of the treated groups compared to the intact surfaces in all the tested materials. More specifically, SEM analysis of the different surface treatments revealed that untreated surfaces (INT) of LV and EN presented a smooth appearance, while BR presented soft grooves upon its surface (Figure 7a]. SH revealed the polished spherical filler particles between some characteristic hole-like round gaps. EN presented a smooth surface with irregular particles due to its ceramic phase. Mechanical roughening treatments (SB and CJ) created a morphological roughened surface, with the presence of irregular craters of different shapes and sizes for BR, LV and EN (Figure 7b,d). SH revealed the same irregular surface along with some visible hole-like round gaps when SB treatment was applied, which were not visible after the CJ treatment application. Chemical conditioning treatment (HF+Si) affected each material differently (Figure 7c). SH exhibited a dissolved surface with spherical hole-like craters in the position of the silica particles, with no strains on its surface. LV exhibited an irregular surface with craters of a different shape to that shown by the mechanical modifications, while the BR showed an area where the amorphous silica particles were dissolved, and potentially undissolved clusters were exposed upon the material surface. In EN, the feldspathic ceramic phase was dissolved superficially, leaving an acupuncture-like irregular surface. 

## 4. Discussion

According to the results of the present study, the first null hypothesis stating that there were no differences among the surface treatments for each CAD/CAM material in surface roughness and morphology should be rejected. Moreover, the second null hypothesis of the study stating that there were no differences in the same surface characteristics among the CAD/CAM materials treated with the same method should also be rejected.

The efficacy of different treatment applications upon the material surface can be described by the surface roughness criteria and are preferable for the S_a_ and S_z_ parameters [25,27] according to ISO 25178. Surface roughness is a component of surface texture, which is quantified by the deviations in the direction of the normal vector of a real surface from its ideal form. In particular, the S_a_ parameter expresses the arithmetical mean height of the surface, while S_z_ is referred to as the maximum average between the highest peaks and highest valleys of the surface [27]. Regarding such polymer materials as composite resins and PICN, surface treatments are recommended to increase the impregnation surface area for resin cements and primers used in dental adhesive procedures [28]. An optical 3D profilometer is able to perform continuous measurements without engraving and damaging the material surface compared to a contact type device. The results of the two methods may vary due to the fact that surface reflections may affect measurement data in the optical 3D method compared to the contact method [29]. 

Micromechanical roughening has been shown to increase bond strength values in CAD/CAM hybrid materials, sometimes even more than chemical conditioning [30,31]. In the present study, micromechanical roughening surface treatments have been applied using two different sandblasting methods (Al_2_O_3_ particles and Cojet Sand) with the same settings in terms of particle size (~30μm) and air pressure (2.5 bar). These parameters were selected in order to avoid superficial cracks upon the material surface and to preserve the homogeneity of the surface treatment [32,33]. 

Chemical conditioning (acid etching) increases the surface roughness and surface energy of the materials, which are necessary for improved micromechanical retention and the wettability of the applied primer leading to optimal bond strength values. It has been found that bond strength values depend mainly upon the interfacial tension between the material and the adhesive, as well as the surface energy of the material itself [34,35]. Even though there is no direct interrelation between surface roughness and surface energy, a higher surface energy leads to higher bond strength values [35]. In the current study, HF acid was used because it is a fluorine-containing etchant which has been shown to produce a roughened surface in most acid-sensitive ceramics and polymers (leucite-based ceramics and silica-based hybrid CAD/CAM materials) [18,30,36]. 

Hydrofluoric acid is colorless, acidic and highly corrosive, and it is in common use in the etching of glass and silicon wafers. It has been reported that, by dissolving and removing the surface layer of a glassy matrix containing silica (SiO_2_), silicates (SiO_4_^−4^) and leucite crystals (K_2_O∙Al_2_O_3_∙4SiO_2_), the surface becomes porous with a pore size of 3–4 μm [37]. The chemical reactions that take place between a siliceous surface and HF are as follows:

4HF + SiO_2_ → SiF_4_ + 2H_2_O (1)

2HF + SiF_4_ → SiF_6_^−2^ + 2H^+^ (2)

SiF_6_^−2^ + 2H^+^ → H_2_SiF_6_ (3)

where HF: hydrofluoric acid, SiO_2_: silica matrix, SiF_4_: silicon tetrafluoride, H_2_O: water, SiF_6_^−2^: hexafluorosilicate, H^+^: hydrogen cation and H_2_SiF_6_: fluorosilicic acid.

Aiming to form a stable bond to PICN, the pre-treatment strategies that should be preferred are HF etching, Cojet system or sandblasting with Al_2_O_3_ particles [38]. Despite the fact that HF application is recommended by the manufacturer of the material and seems to develop favorable bond strength values, it has been shown that sandblasting with Al_2_O_3_ particles followed by silane application can also achieve optimal bond strength [31,39]. Indeed, in the present study, the only significant differences regarding S_a_ values were found between HF + Si and CJ, suggesting that these two treatments seem to be ideal for this material (EN)—a finding which coincides with other studies [18,31]. The highest S_a_ values were found after treatment with CJ, whereas the highest S_z_ values were found after sandblasting with Al_2_O_3_. Although the differences are significant for S_a_ units, the clinical significance regarding bond strength values is questionable [29]. 

When EN is used as a restorative material, sandblasting procedures should be done with a maximum particle size of 50 μm, since crack formation has been observed with larger-sized particles (120 μm), while the duration of the procedure should not exceed 30 s [40]. Strasser et al. [29] found that sandblasting with 50 μm Al_2_O_3_ at approximately 2 bar of air pressure produced an average increase of 225% in surface roughness (R_a_) values without creating any serious surface defects, while Tecke et al. [40] reported that over 30 s of sandblasting duration, superficial cracks are formed that may expand up to 3 μm into the material mass. This may be related to the fact that EN presents a high feldspar ceramic phase and thus contains a lower polymeric phase compared to composite CAD/CAM blocks. This leads to a more susceptible material to HF + Si treatment, resulting to higher S_z_ values than composite materials.

Various surface treatments have been proposed for bonding on CAD/CAM composite materials [30]. Depending on the composition of each material, HF etching dissolves the ceramic components, while sandblasting creates the roughened surface in order to increase the crucial for bonding, micromechanical retention. Silane coupling agents may enable adhesion to the different kinds of substrates of these materials; therefore they improve the bond strength values by priming the inorganic components of the hybrid composite blocks embedded in the resin matrix [36]. BR contains SiO_2_ and barium (Ba) glass particles which have different interaction with HF etching. In particular, during HF treatment the SiO_2_ particles are dissolved forming a desirable roughened surface but Ba glass particles might not be completely dissolved and thus, the use of a suitable material primer, which also reacts with Ba particles, may lead to a better adhesion surface [41]. SEM images revealed the exposure of Ba glass particles upon the surface of the material after HF acid etching, and this might explain that S_z_ values of BR after HF + Si treatment were significant lower compared to the other materials. SH contains large spherical particles, which seem to be rather susceptible to HF treatment since there was a clear loss of substance with hole-like gaps at the filler sight. This might be attributed either to the low filler mass (61%), or to the gaps which may be pre-existing upon the material surface due to its fabrication method according to SEM evaluation. SH presented significant lower S_z_ values when treated with HF + Si compared to SB and CJ treatments, a fact which may be attributed to the plane surface remained after the treatment. It has been demonstrated [18] that HF + Si had a different effect on LV, since it had a lower influence on dissolving the glassy phase of the material surface and only small gaps and micropores were created. As a matter of fact, the S_z_ value of the material after HF + Si was significantly lower compared to the SB and CJ surface treatments. 

Mechanical roughening treatments such as those used in this study (SB and CJ) have been shown to create a coarse surface and enhance the bond strength values of composite CAD/CAM materials [31,42]. In terms of surface roughness parameters, SH showed significantly higher Sz values compared to LV and BR when treated with SB and CJ—a fact which may occur due to the relatively lower filler mass of SH (61%) and the easier detachment of the filler particles due to the loose bonding with the resin matrix [32]. LV has higher filler content compared to the other composite materials tested in this study, with smaller fillers and larger cluster particles which cannot easily be detached from the material surface during sandblasting procedures, thus presenting significant lower S_a_ values compared to SH and BR as seen in other studies [43]. This can also explain the fact that LV presented significant differences between mechanical and chemical surface roughening methods for both S_a_ and S_z_. BR exhibited the same behavior as LV after the micromechanical surface treatments, which may be due to similarities in their chemical structure.

Regarding the limitations of the present study, it must be noted that all the experimental procedures took place under ideal laboratory conditions. Restorations which are manufactured out of CAD/CAM blocks do not present a flat surface as with the control group (INT) of the present study; in contrast, they represent the reflection of the prepared dental surface which might have angular surfaces or indentations. This means that, on the one hand, sandblasting procedures are never accurate regarding distance and tip angle placement, while on the other hand, surface chemical conditioning with HF might present an uneven distribution upon the material surface.

## 5. Conclusions

Within the limitations of the present study, it could be concluded that in all the tested materials, surface treatments resulted in higher surface roughness values compared to the control groups. Among the surface treatments, there were negligible differences that may not have clinical significance. Different treatments affected the surface properties of the materials, presumably due to discrepancies in their composition and structure. Further research is needed to correlate the surface properties of the materials after the treatments with the bond strength to the tooth tissues.

## Figures and Tables

**Table 1:** The technical characteristics of the tested computer-aided design/computer-aided manufacture (CAD/CAM) materials.

Material	Manufacturer	Type	Composition	Lot Number
Shofu Block HC	Shofu Inc, Kyoto, Japan	Composite	UDMA, TEGDMA, silica (20 nm), barium glass (300 nm), silica powder, micro fumed silica, zirconium silicate	111501
Brilliant Crios	Coltene AG, Altstätten, Switzerland	Composite	Bis-MEPP, UDMA, DMA, amorphous SiO_2_ (<20 nm), barium glass (<1 nm)	I81413
Lava Ultimate	3M ESPE, Seefeld, Germany	Composite	Bis-GMA, UDMA, Bis-EMA, TEGDMA, SiO_2_ (20 nm), ZrO_2_ (4–11 nm), aggregated ZrO_2_/SiO_2_ cluster (SiO_2_: 20 nm, ZrO_2_: 4–11 nm)	N721283
Vita Enamic	Vita Zahnfabrik, Bad Sackingen, Germany	Hybrid ceramic (PICN)	UDMA, TEGDMA (14%), Feldspathic crystalline particles in glassy matrix	80670

Bis-GMA: Bisphenol A diglycidylmethacrylate; Bis-EMA: Ethoxylated bisphenol A dimethacrylate; Bis-MEPP: 2,2-bis(4-methacryloylethoxyphenyl) propane; DMA: dimethacrylate; TEGDMA: triethylene glycol dimethacrylate; UDMA: urethane dimethacrylate; Al_2_O_3_: aluminum oxide; SiO_2_: silica, ZrO_2_: zirconia.

**Table 2:** Means and standard deviations of S_a_ (μm) and S_z_ (μm) with respect to material and surface treatment. The same lowercase superscripts in columns indicate no statistically significant difference (*p* > 0.05).

Sources		Group Size (n)	Dependent Variables			
Material	Surface Treatment		S_a_		S_z_	
			Mean	Standard Deviation	Mean	Standard Deviation
	INT	8	0.192^a^	0.005	1.712^a^	0.435
SH	SB	8	0.225^b^	0.002	6.037^b^	0.709
	HF + Si	8	0.222^b,c^	0.005	4.587^c^	0.699
	CJ	7	0.218^c^	0.003	6.842^b^	0.229
	INT	8	0.182^d^	0.007	1.000^d^	0.292
LV	SB	8	0.219^c^	0.004	5.787^b^	0.825
	HF + Si	7	0.228^e^	0.001	2.171^e^	0.423
	CJ	7	0.222^c^	0.003	5.485^b^	0.625
	INT	8	0.184^a,d^	0.009	0.475^f^	0.158
BR	SB	8	0.228^e^	0.003	5.925^b^	1.188
	HF + Si	8	0.227^e^	0.001	1.287^e^	0.699
	CJ	8	0.227^e^	0.002	5.275^b^	0.837
	INT	7	0.196^a^	0.001	2.885^f^	0.705
EN	SB	6	0.222^c,b^	0.005	6.300^b^	0.363
	HF + Si	8	0.217^c^	0.003	5.900^b^	0.607
	CJ	7	0.225^b^	0.003	5.742^b^	0.113

SH: Shofu Block HC, LV: Lava Ultimate, BR: Brilliant Crios, EN: Vita Enamic, INT: intact, SB: sandblasting, HF + Si: hydrofluoric acid and silane, CJ: Cojet system.

**Table 3:** Full factorial analysis of variance results (exact significance levels).

Sources	Dependent Variables	
	S_a_	S_z_
*Main effects*		
Material	0.031	0.000
Surface treatment	0.000	0.000
*Interaction effects*		
Material × surface treatment	0.000	0.000
